# Investigation of hindered phenol antioxidant effects on the aging performance of cross-linked LDPE in the presence of copper

**DOI:** 10.1038/s41598-020-67131-1

**Published:** 2020-06-23

**Authors:** Jianxi Li, Cheng Zhou, Siyi Xu, Liguo Shen

**Affiliations:** 1 National Energy Life Evaluation and Management Technology Lab of Nuclear Power and Nonmetal Materials, Suzhou, 215400 PR China; 2 CGN-DELTA (Taicang) Testing Technology Co., Ltd., Suzhou, 215400 PR China; 3 College of Geography and Environmental Sciences, Zhejiang Normal University, Jinhua, 321004 PR China

**Keywords:** Materials chemistry, Polymer chemistry

## Abstract

In this study, LDPE materials with different kinds of antioxidants were prepared by melt-blending method. To reveal the aging mechanism and the anti-oxidation efficiency of LDPE in the presence of copper, series of characterizations including tensile testing, Fourier transform infrared spectra (FTIR), differential scanning calorimetry (DSC), scanning electronic microscopy (SEM) and computation simulation were performed. The experimental results indicated that the aging process significantly decreased the tensile strength and elongation of those aged samples except 1024, which retarded the aging degradation of LDPE at the same condition. These results were further confirmed by the FTIR analysis with the carbonyl index values. Additionally, the melting peaks of DSC plots became broader and shifted to the lower temperatures during the aging process for S-0, S-3114 and S-1010, whereas no obvious changes were observed for S-1024. Importantly, according to the results obtained from computation simulation, a strong metal-ligand interaction between hydrazide group and copper ions was formed to prevent the further oxidation, which accounted for the excellent anti-oxidation behavior of 1024 for LDPE in the presence of copper.

## Introduction

Comparing with general organic polyolefin, polyethylene (PE) has excellent temperature resistance, electrical insulation, dielectric properties and weather resistance due to its long chains with regular CH_2_ units. Over the past decades, PE materials have been widely used in various areas, such as medical and health, aerospace, wire and cable, automotive industry, architecture and so on^1–4^. Unfortunately, it was found that the chemical structure and composition of PE can be significantly destroyed as a result of the comprehensive factors including crosslinking byproducts, metal impurities, ionic impurities, electrical and mechanical effects, moisture, and chemical substances, which result in the failure of the insulation performance^5,6^. For example, it has been reported that thermal aging can lead to the deterioration of electrical and mechanical properties of PE insulation^3,7,8^.

As discussed above, the introduced metal impurities by the process of manufacturing or the operating can lead to the degradation or even failure of PE insulation^3^. The metallic impurities consisting of copper powders, originated from the conductor or the ground water, could form heterocharges, which play a role of enhancing the electric stress and promoting the initiation of water tress, and thus decreasing the strength of the PE insulation. As claimed by Gillen^9^, copper powders could diffuse in ethylene propylene rubber(EPR) insulation matrix during the extrusion process, which can account for the degradation of insulation materials during the thermal aging. The introduction of antioxidant into hydrotalcite can shorten the oxidation induction time, which was indicative of the protective action towards oxidation of polymer, whereas, the impurity of hydrotalcite and metal ions can increase the rate of polypropylene photo oxidation^10^. Importantly, copper conductor plays an important role in the use of cables, therefore, it is of great significance to meet its requirements in anti-oxidation environments by solving the catalyze effect of copper on the aging performance of PE insulation^3,6,11,12^.

Blending of antioxidant is usually regarded as a simple and effective way to improve the performance of PE materials and the long-term service ability^13–17^. Additionally, the introduced antioxidant protects the polymers from thermal aging damage by blocking the reactions of free radical with molecular oxygen^18^. Phenol-type antioxidants including the hindered phenolic compounds are the most important classes of antioxidants being extensively used because of their antioxidant activities with a small content^4,19^. The excellent performance of hindered phenolic antioxidants is closely related to its electrical delocalization, relative molecular weight, structures, the number and type of the substituents^20^.

Therefore, an antioxidant with the ability of hindering the copper catalysis on PE insulation aging performance should be emphasized. In this study, three kinds of hindered phenolic antioxidants with different structures were used to investigate its effects on the aging performance of PE in the presence of copper powders. The structure variations and aging properties were measured by Fourier transform infrared spectrometer (FTIR), tensile test, differential scanning calorimetry (DSC) and other equipment. Finally, the aging mechanism was analyzed and further confirmed by analog computation.

## Materials and Methods

### Materials

Low density polyethylene (LDPE), C410 (MFI: 3.5 g/10 min; Density: 0.918 g/cm^−3^), was provided by UBE Industries, Ltd., Japan. Copper powder was supplied by Luohe HTP Advanced Material CO., Ltd., Henan, China. Hindered phenolic antioxidants 1024, 3114 and 1010 were provided by Qianchang Tradeing Co., Ltd.

### Sample preparation

The samples were prepared by mixing LDPE, copper powder and antioxidants (Fig. S1) with certain ratios as given in Table 1. Firstly, all the materials were mixed in the open mill at 90 °C; Secondly, to obtain polymer sheets, all the samples were laminated in a curing press at 175 °C for 5 min. Then, the laminates were cut into bars according to the corresponded national standard. At last, all the samples were cross-linked by electron beam irradiation method (Energy of electron accelerator: 1.2 M eV; Irradiation dose: 120 kGy; Radiation dose rate: 2.0 × 10^5^ kGy/h). Afterwards, thermal aging treatment of these samples was conducted in an oven at 150 °C for varied times before examined (0 h, 24 h, 48 h and 72 h) in air.

**Table 1:** Composition of the aged samples with different antioxidants.

Sample	LDPE (g)	Cu (g)	Antioxidants (g) ^1^		
			1024	3114	1010
S-0	100	20	0	0	0
S-1024	100	20	0.20	0	0
S-3114	100	20	0	0.28	0
S-1010	100	20	0	0	0.42

1) the molar ratio of antioxidants with LDPE was 0.00036 mol.

### Characterization

Tensile testing: Mechanical performance of the aged samples were carried out with a crosshead speed of 250 mm/min by universal testing machine (CMT4104, MTS, China) at 25 °C. The dimensions of these specimens were 75 mm × 4 mm × 1 mm.

Scanning electron microscope (SEM): The morphology and elemental analysis investigation were carried out using a SEM-EDS equipment (scanning electron microscope with energy-dispersive spectrometry, Phenom Pro, Phenom-World B.V. Company, Netherlands). The electron beam voltage for the observation was 15 kV. The samples were thickness sheets of 1.5 mm. The samples were sputter-coated with gold before analysis.

Differential scanning calorimetry (DSC): crystallization and melting temperatures, crystallization enthalpy and the crystallization degree were measured by Q20 (TA, USA) differential scanning calorimeter. Each sample was heated to 130 °C at the rate of 10 °C/min, keeping 2 min to remove the thermal history, then cooled to 30 °C at the rate of 10 °C/min, holding 2 min and finally reheated to 130 °C with the same rate.

Fourier transform infrared spectra (FTIR): The spectra of the aged samples were collected in reflection model at a resolution of 4 cm^−1^ and 16 scans from 4000 to 500 cm^−1^ with the TENSOR II instrument (Bruker, Germany).

Computation simulation: the quantum chemical computations were carried out using the GAUSSIAN03 suite of programs at the B3LYP level in conjunction with the SDD and 6–31 G** basis set for copper ions and other atoms, respectively, and 6–311 G** basis set was used to processed the high-precision calculation. Besides, the carbonyl was regarded as the stationary points by frequency calculations. Gibbs free energies were computed using the rigid-rotor harmonic approximation implemented in GAUSSIAN03. To simulate the kinetic process between the polymer and copper ions at the temperature range from molten state to the room temperature, a semi empirical molecular dynamics calculation with XTB was performed. Additionally, this method was a simplified model. In this way, only the combination process of monomer and copper ions was taken, and no more analysis was conducted because the binding site can meet the calculation requirements in the process of the qualitative analysis.

## Results and discussions

### Dispersion of copper

**Figure 1:**
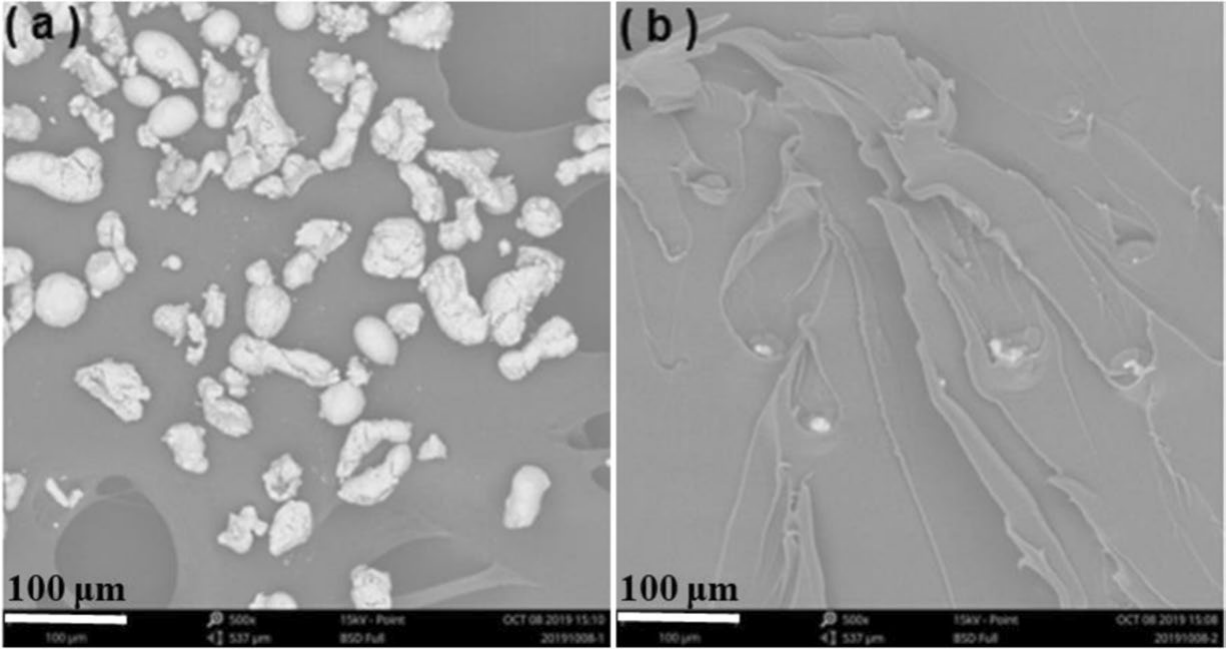
SEM images of copper particles and their dispersion.

Generally, the inherent properties of copper powders and their dispersion in matrix determining the relative performance of LDPE. As shown in Fig. 1(a), large numbers of copper powders with relative smooth surface and irregular shapes could be observed on the surface of the conductive adhesive. The sizes of those copper powder were in the range of micron magnitude, which meant a more stable activity in comparison with nanoparticles. Therefore, agglomeration will be observed in LDPE/copper materials. Figure 1(b) described the dispersion of copper powders in LDPE matrix. The white particles distributed on the internal areas of LDPE as shown in SEM image.

### Mechanical performances

**Figure 2:**
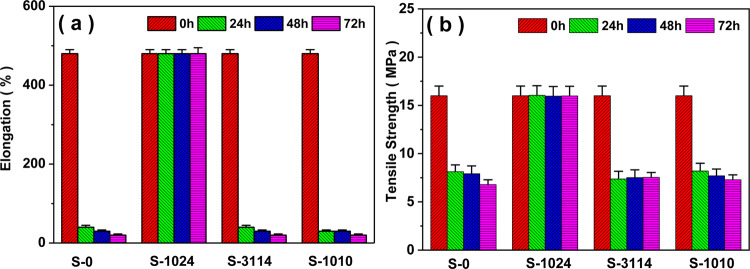
Influence of antioxidants on mechanical properties of the aged samples.

The effects of aging time and hindered phenolic antioxidants on mechanical properties of LDPE materials before and after thermal oxidative aging were shown in Fig. 2. As shown in Fig. 2(a), samples with 1024 had higher result after aging process compared to other aged samples. But the graph showed a significant decrease of the tensile strength and elongation at break of these aged samples except S-1024. For S-1024, the elongation at break of these unaged samples was almost unchanged with a value of 480% regardless of aging time. However, with the aging time reached to 72 h, the values of LDPE, S-3114, S-1010 decreased to 40%, 30% and 30%, respectively, owing to the degradation of LDPE after hot-air aging in the presence of copper powder, whereas that of S-1024 remained constant. The tensile strength showed the relative similar trend as shown in Fig. 2(b). At 72 h, the values of LDPE, S-3114, S-1010 displayed an obvious decline by 56.3%, 55.8% and 57.0%, respectively, while that of S-1024 remained constant. It was reported that formation of a complex combination between metal ions and organic molecules could result in a reduction or an enhancement of antioxidant properties^21^. Thus, it is supposed that the complex combination between 1024 and Cu^2+^ made a contribution to the anti-oxidation effectiveness for LDPE as a result of the specific metal-ligand interactions.

### Structure analysis

To further understand the aging mechanism and the efficiency of these hinder phenolic antioxidants, all the aged samples were analyzed by FTIR. Fig. S2 gave the IR spectra of the carbonyl absorption region of these aged samples with different antioxidants at varied times. As shown in Fig. S2(f), the intense bands of LDPE were located at 2916 and 2847 cm^−1^, which were assigned to the asymmetric and symmetric absorption of the CH_2_ groups, respectively, while those at 1472 and 730 cm^−1^ were corresponded to the bending stretching and the inner rocking vibration of CH_2_ groups, respectively^13,22^. After aging, new absorption peaks appeared at the shift of 1726 and 1600 cm^−1^, which were attributed to the stretching vibration of C = O and C = C bonds, respectively^23^. As shown in Fig. S2, no obvious peaks were found in the spectra of S-1024, which suggested that 1024 possessed the best antioxidant efficiency in LDPE materials. With the aging time increased, the intensity of peaks at 1726 and 1600 cm^−1^ increased, while that of CH_2_ at 2916 and 2847 cm^−1^ displayed the down trend, which were indicative of the aging of LDPE and the less efficiency of antioxidants 3114 and 1010. The thermo-oxidation degradation rate of polymer is generally determined by the content of oxygen and free radicals. During the degradation process, free radicals could be produced due to the breakage of chemical bond of LDPE in the atmosphere of light, heat, mechanical force and other factors. To intuitive describe the aging degree of LDPE and the compared anti-oxidation effect of these antioxidants, carbonyl index (*CI*) was incorporated as following:

1 $$CI=\frac{Ab{s}_{1726c{m}^{-1}}}{Ab{s}_{1472c{m}^{-1}}}\times 100 \% $$

where $Ab{s}_{1726c{m}^{-1}}$ and $Ab{s}_{1472c{m}^{-1}}$ are the absorption intensity of C=O and CH_2_ peaks, respectively. Figure S2(e) displayed the varied *CI* values of these aged samples. The *CI* values of S-1024 increased to 9.2% with the aging time increased to 72 h in comparison with LDPE, S-3114 and S-1010, that of the latter aged samples were 128.1%, 128.8% and 168.9%, respectively. In terms of *CI*, the values of LDPE, S-3114 and S-1010 were almost 14 and 18 times larger than that of S-1024, which implied that 1024 had the better anti-oxidation effect in the presence of copper powder. There was an agreement with the result of tensile test. Besides, at the same aging time, S-3114 and S-1010 had the higher *CI* values in comparison with LDPE.

### Melting behaviors

**Figure 3:**
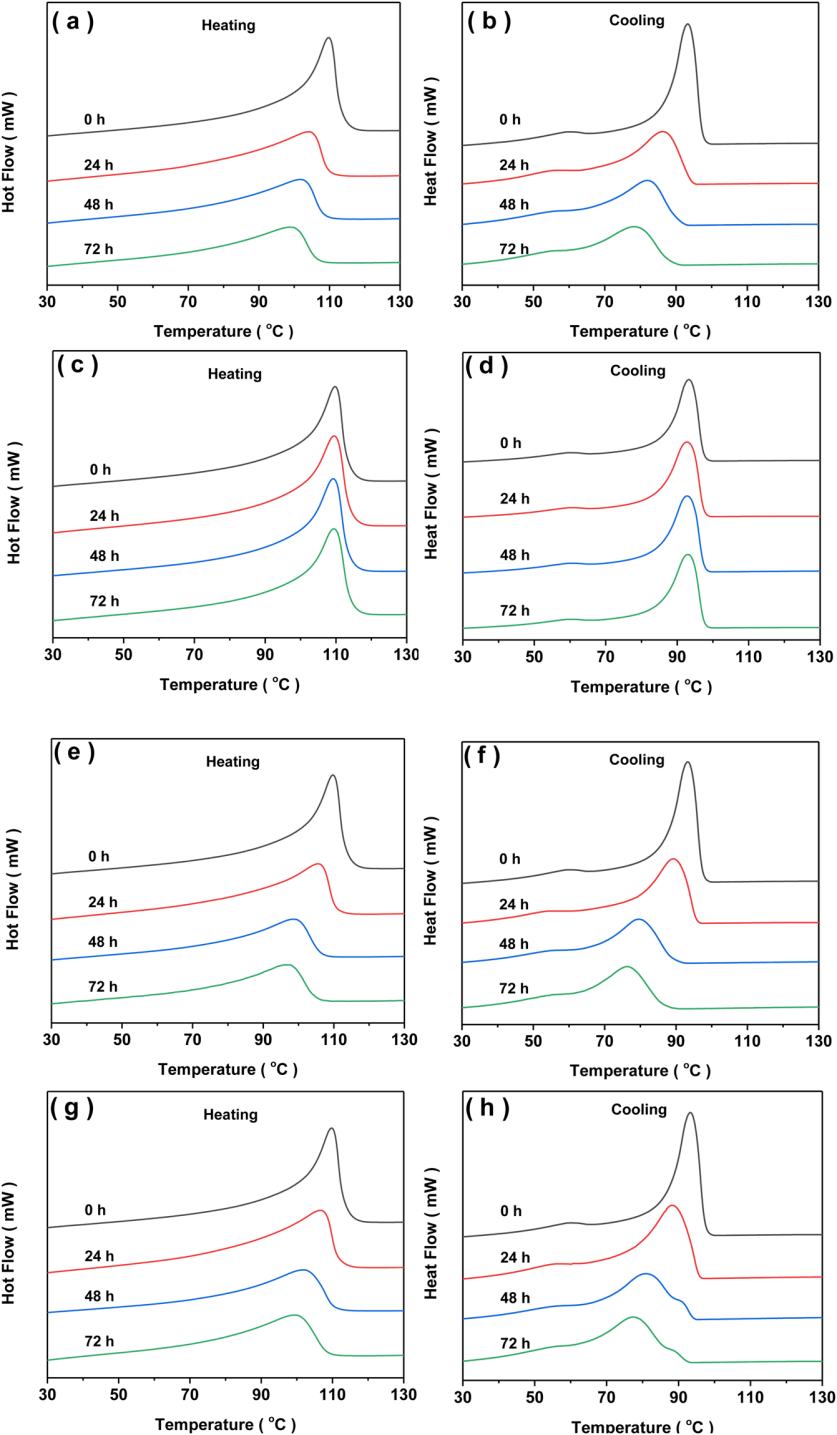
Melting and cooling plots of the aged samples with different antioxidants (S-0(**a, b**); S-1024(**c, d**); S-3114(**e, f**); S−1010(**g, h**)).

To understand the role of antioxidants in the performances of LDPE in the presence copper powder, it is important to investigate the thermal ability of these aged materials. All DSC procedures were conducted for S-0, S-1024, S-3114 and S-1010 by recording the melting point, crystallization point, melting enthalpy, crystallinity degree as shown in Tables 2–5. Figure 3 showed the DSC cooling and subsequently heating traces of the aged samples with different antioxidants. As shown in Fig. 3, LDPE presented sharper crystallization and melting peaks, however, with the increase of aging time, these peaks became broader and shifted to the lower temperatures, which indicated that the regular crystalline of LDPE was significantly disturbed by the breakup of the long CH_2_ chains due to the degradation of LDPE via hot-air aging^24^. DSC results revealed that the formed crystal defects including oxygenated groups and double bonds during oxidative degradation process resulted in the decline of the melting and crystallization temperatures. Compared with S-0, S-3114 and S-1010, the melting temperature, the crystallization temperature and the shapes of peaks of S-1024 were almost constant regardless of aging temperature, which meant a better anti-oxidation of 1024.

**Table 2:** Results of S-0 obtained from DSC plots.

Aging time (h)	Onset (°C)	Peak (°C)	∆H (J/g)	T_c_ (°C)	X_c_ (%)
0	98.7	109.7	98.8	93.3	36.2
24	77.6	104.5	83.1	86.4	30.4
48	71.1	101.7	68.8	82.2	25.2
72	67.9	98.9	63.6	78.6	23.3

**Table 3:** Results of S-1024 obtained from DSC plots.

Aging time (h)	Onset (°C)	Peak (°C)	∆H (J/g)	T_c_ (°C)	X_c_ (%)
0	98.2	109.2	99.6	93.0	36.5
24	97.8	109.3	99.8	92.5	36.6
48	98.1	109.2	102.1	92.6	37.4
72	98.0	109.5	102.0	92.7	37.4

**Table 4:** Results of S-3114 obtained from DSC plots.

Aging time (h)	Onset (°C)	Peak (°C)	∆H (J/g)	T_c_ (°C)	X_c_ (%)
0	99.0	109.7	98.8	93.1	36.2
24	82.3	105.5	83.1	89.2	30.4
48	72.7	98.7	66.3	79.8	24.3
72	70.1	96.2	62.3	76.7	22.8

**Table 5:** Results of S-1010 obtained from DSC plots.

Aging time (h)	Onset (°C)	Peak (°C)	∆H (J/g)	T_c_ (°C)	X_c_ (%)
0	99.8	109.8	99.1	93.0	36.3
24	80.2	106.6	87.1	88.3	31.9
48	73.0	101.6	70.0	81.1	25.6
72	69.8	99.5	65.1	77.5	23.8

Furthermore, crystallinity of these aged samples was calculated by the equation as following:

2 $${X}_{c}=\frac{\Delta {H}_{m}}{\Delta {H}_{0}}\times 100 \% $$

where the ${X}_{c}$, $\Delta {H}_{m}$ and $\Delta {H}_{0}$ were the crystallinity, melting enthalpy and enthalpy for the complete crystallization, respectively. According to Ref 25,the value of $\Delta {H}_{0}$ for LDPE was 273.0 J/g.

The melting temperature, crystallization temperature and the crystallinity of S-0 were 109.7 °C, 93.3 °C and 36.2%, respectively, while that of S-0 decreased to 98.9 °C, 78.6 °C and 23.3%, respectively, with the aging time increased to 72 h, which were attributed to the unregularly lamellar crystallinity packing as a result of the degradation of LDPE chains during aging process^26–28^. It was reported that the thermo-oxidative aging process could be divided into the physical aging stage and the chemical aging stage in PE aging process. As illustrated by Li^29^, at aging 150 °C, chemical aging happened. Thus, the thermo-oxidative aging increased the lamellar spacing and the destruction of spherulites, which resulted in the decrease in crystallinity. The same trend could be found for S-3114 and S-1010. The relative parameters of melting and crystallization behaviors of S-1024 almost remained constant in comparing with other aged samples. Thus it was notable that the catalysis of copper ion was retarded by 1024 via the metal-ligand interactions.

### Anti-oxidation mechanism

**Figure 4:**
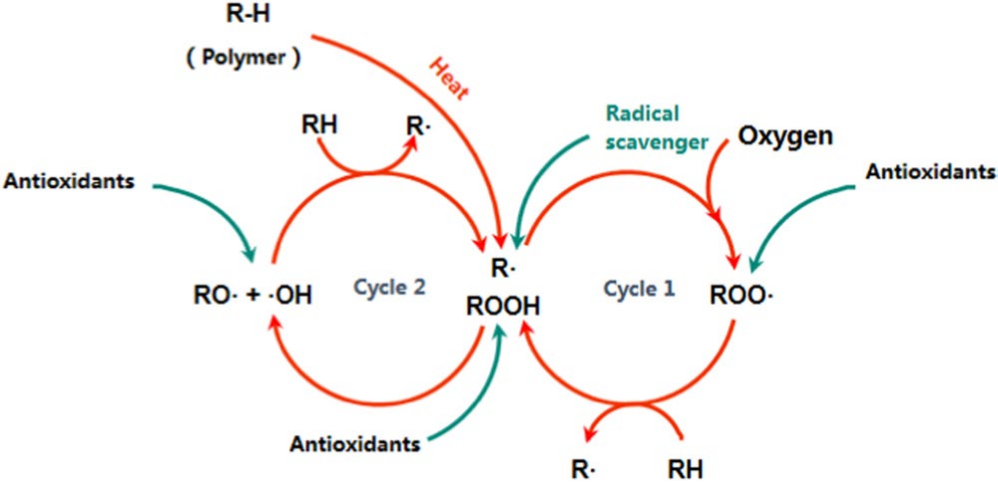
Anti-oxidation mechanism of antioxidant in aging process of LDPE.

The oxidation mechanism of LDPE and the anti-oxidation effect of antioxidant in the period of aging were described in Fig. 4. Oxidation of many polymers occurs by a free‐radical, thus it was of great importance to realize the actual oxidation process of LDPE/antioxidants materials in the presence of copper. As shown in Fig. 4, the red lines on behalf of the oxidation process while the green lines represented the anti-oxidation mechanism of antioxidants. During the process of oxidative aging, main components including perhydrates, alkyl/alkoxyl radicals and the hydroxyl radicals should be emphasized^15^. Chain segments were oxidized to generate the perhydrates, which can be decomposed into free radicals. Then hydrogen atoms from other chains were captured by these free radicals, leading to the generation of other perhydrates and free radicals. All the perhydrates and free radicals above participated into oxidation reactions in turn and kept the process successive^30,31^. The hydrogen atoms of phenolic hydroxyl have the higher activity in comparing with other atoms of hindered phenolic antioxidants, which would react with the free radicals to terminating the oxidation reaction. It was reported that copper can act as a catalyst in oxidation reaction in polymer, which can accelerate the generation of perhydrates as following procedure^3,32^.

3 $${\rm{ROOH}}+{{\rm{Cu}}}^{+}\to {\rm{RO}}+{{\rm{HO}}}^{-}+{{\rm{Cu}}}^{2+}$$

4 $${\rm{ROOH}}+{{\rm{Cu}}}^{2+}\to {\rm{ROO}}+{{\rm{H}}}^{+}+{{\rm{Cu}}}^{+}$$

**Figure 5:**
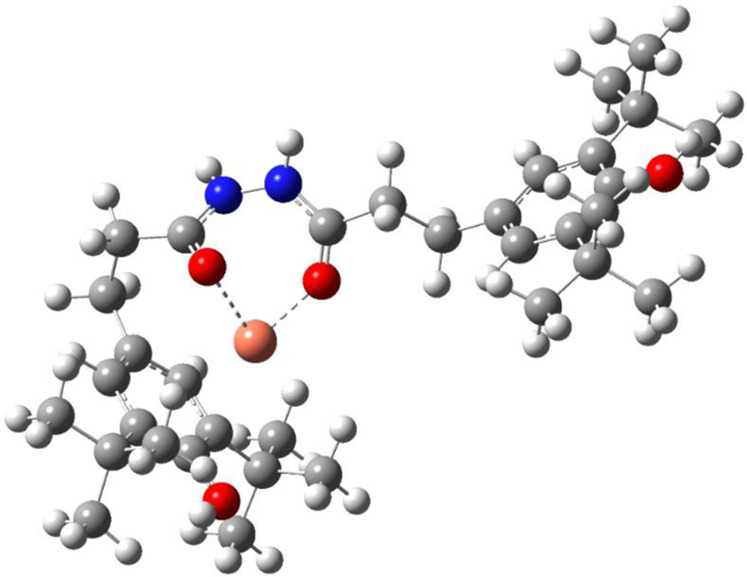
Complex conformation structures between 1024 and copper ions obtained from DFT calculations.

As discussed above, 1024 shown the best anti-oxidation effect in the presence of copper in comparison with 3110 and 1010 for LDPE, which was attributed to the metal-ligand interactions between hydrazide group and copper ions to prevent the further oxidation of materials^10,33^. Therefore, computation simulation was used to confirm the existence of complex. To obtain the conformation distribution of the complex, XTB was used to perform the semi-empirical molecular dynamics calculations by simulating the kinetic process of the interaction between copper ions and 1024 molecule in the temperature ranges from its molten state to room temperature. The conformation structure of the complex obtained from DFT calculation was described in Fig. 5.

As shown in Fig. 5, the red, blue, win red, dark grey and light grey balls belong to the oxygen, nitrogen, copper, carbon and hydrogen atoms, respectively. It was obvious that the complex effect was reflected by the interaction between oxygen atom of hydrazide and copper ions. The larger the bond energy was, the more stable the intensity of complex was. The complex bond energy obtained from simulation was 470.6 kcal/mol, which was indicative of the stability of metal-ligand interactions. Above all, all these results illustrated that 1024 possessed the optimal anti-oxidation effect for LDPE in the presence of copper powders during the thermal aging process.

## Conclusions

In this study, the impacts of aging time and the anti-oxidation effect of these antioxidants on the performances of LDPE in the presence of copper were investigated. As revealed by tensile testing results, for S-1024, the relative tensile parameters remained constant regardless of aging time due to the strong interaction of the complex formed, which suggested that 1024 possessed the certain anti-oxidation effects in the presence of copper. The aging process was traced by FTIR and the carbonyl index was introduced to intuitive illustrated the oxidation behaviors of LDPE with different antioxidants. With the aging time increased, the intensity of peaks at 1726 and 1600 cm^-1^ increased, which were indicative of the oxidation aging of LDPE and the less efficiency of 3114 and 1010 during the degradation process. Besides, compared with S-1024, all the relative parameters of melting and crystallization behaviors of S-0, S-3114 and S-1010 were decreased with the increasing aging time due to the thermal oxidation behaviors. The accelerated thermal oxidation aging process was hindered by 1024 as a result of the formation of complex between copper ions and the corresponding antioxidant in combination with the constant values obtained from DSC traces. Importantly, a rather higher complex bond energy (470.6 kcal/mol) was obtained by computation simulation, which was indicative of the stability of metal-ligand interactions as a favorable evidence for the formation of complex. The complex interaction limited the catalysis of copper during the aging process, which also explained the excellent anti-oxidation efficiency of 1024.

## Supplementary information

Supplementary Information.

Supplementary Information.
