# Supplementary material for: Investigation of hindered phenol antioxidant effects on the aging performance of cross-linked LDPE in the presence of copper
Source: Sci Rep. 2020 Jun 23;10:10189. doi: 10.1038/s41598-020-67131-1 (PMC7311468; doi:10.1038/s41598-020-67131-1)
Supplement: Supplementary file 1 — Supplementary Information. [file 41598_2020_67131_MOESM1_ESM.docx]

**Investigation of hindered phenol antioxidant effects on the aging performance of cross-linked LDPE in the presence of copper**

Jianxi Li ^1^ Cheng Zhou ^2^ * Siyi Xu ^3^ Liguo Shen ^3^

1 National Energy Life Evaluation and Management Technology Lab of Nuclear Power and Nonmetal Materials, Suzhou, 215400, PR China

2 CGN-DELTA (Taicang) Testing Technology Co., Ltd., Suzhou, 215400, PR China

3 College of Geography and Environmental Sciences, Zhejiang Normal University, Jinhua, 321004, PR China

Fig. S1 Chemical structures of antioxidants

Fig. S2 Influence of antioxidants on structures of the aged samples
